# Morphology and Electric Conductance Change Induced by Voltage Pulse Excitation in (GeTe)_2_/Sb_2_Te_3_ Superlattices

**DOI:** 10.1038/srep33223

**Published:** 2016-09-13

**Authors:** Leonid Bolotov, Yuta Saito, Tetsuya Tada, Junji Tominaga

**Affiliations:** 1 Nanoelectronics Research Institute, National Institute of Advanced Industrial Science & Technology (AIST), Tsukuba 305-8565, Japan

## Abstract

Chalcogenide superlattice (SL) phase-change memory materials are leading candidates for non-volatile, energy-efficient electric memory where the electric conductance switching is caused by the atom repositioning in the constituent layers. Here, we study the time evolution of the electric conductance in [(GeTe)_2_/(Sb_2_Te_3_)_1_]_4_ SLs upon the application of an external pulsed electric field by analysing the structural and electrical responses of the SL films with scanning probe microscopy (SPM) and scanning probe lithography (SPL). At a low pulse voltage (1.6–2.3 V), a conductance switching delay of a few seconds was observed in some SL areas, where the switch to the high conductance state (HCS) is accompanied with an SL expansion under the strong electric field of the SPM probe. At a high pulse voltage (2.5–3.0 V), the HCS current was unstable and decayed in a few seconds; this is ascribed to the degradation of the HCS crystal phase under excessive heating. The reversible conductance change under a pulse voltage of opposite polarity emphasised the role of the electric field in the phase-transition mechanism.

Phase-change materials based on Ge, Sb and Te have long been used in optical memory devices such as DVDs and are leading candidates for non-volatile electric memory such as phase-change random-access memory (PC-RAM). It was found that superlattice films consisting of periodic nanometre-thick layers of GeTe and Sb_2_Te_3_ are a new class of two-dimensional (2D) crystals that possess unique physical properties - giant magnetoresistivity and magneto-optical effects, without magnetic impurities^1^,^2^,^3^. In traditional Ge-Sb-Te alloys, the conductance switching is driven by a thermally activated transition between the amorphous and crystalline states^4^,^5^,^6^. In contrast, the switching in SL films requires an order of magnitude of less energy than the traditional process, and it occurs in a much shorter time^7^, indicating that another mechanism of the crystal-to-crystal phase transition involving the repositioning of atoms in the crystal layers is responsible^8^.

There have been significant efforts in the fabrication of novel non-volatile memory cells^9^,^10^,^11^ and ultra-fast optical elements^12^ incorporating SL films as recording media and in theoretical work to gain understanding of the mechanism underlying the crystal-to-crystal phase transition at an atomic level^13^,^14^,^15^. At present, there is agreement by different groups that under an electric field applied normally to the crystal layers, conductance switching is caused by the displacement of the Ge atom between sites with rhombohedral and octahedral symmetries. Two competing atomic models of the SL stack were proposed to describe the high electric conductance state (HCS). One structural model consists of a Ge-Te- Ge-Te stack with a ferroelectric GeTe order in the HCS due to a Te-Ge-Te bond flip along the c-axis of the 2D crystal, called a Ferro model in ref. 14. This structure is predicted to be the most stable configuration above 125K^15^. Another model assumes that the switching involves a Te-Ge-Te bond flip in two adjacent atomic layers in opposite directions, called a Petrov model^9^. Early experimental studies using scanning probe microscopy (SPM) and X-ray diffraction spectroscopy have confirmed the crystalline states of chalcogenide films such as GeSb_2_Te_4_ in ref. 16 and Sb_2_Te_3_ in ref. 17, prepared by sputtering at a Si substrate temperature of 230 °C. Recently, a detailed study of the interface formation in [(GeTe)(1 nm)/(Sb_2_Te_3_)(3 nm)]_15_ SLs using high-angle annular dark field scanning transmission electron microscopy (HAADF-STEM) has shown SL grains with mixed Ge/Sb atomic layers prepared by molecular beam epitaxial growth on the Sb-terminated surfaces of Si(111)^18^,^19^. Because the mixing of Ge/Sb atomic layers changes the local stacking in the SL grains, a study of position-dependent electric properties in such granular SL films is crucial to extending our understanding of both the conductance state transition and the performance variations in scaled SL memory cells.

Here, we report on the local conductance state switching in [(GeTe)_2_/(Sb_2_Te_3_)_1_]_4_ SLs by employing scanning probe microscopy (SPM) and scanning probe lithography (SPL). SPM is a powerful technique for investigating the conductance states of solid films because of its high spatial resolution. SPL has emerged as a type of lithography for academic research, and it combines the ability to create nanoscale features and to handle soft matter such as proteins and polymers^20^. Therefore, SPL patterning of SL films is an ideal way to investigate the position-dependent conductance switching in SL films without introducing mechanical stress from a metal electrode. Using SPM and SPL, we examined the [(GeTe)_2_/(Sb_2_Te_3_)_1_]_4_ SL, as it has the simplest SL unit cell structure consisting of (GeTe)_2_/Sb_2_Te_3_ units. By holding a sharp metal probe at a predefined distance from the SL surface [see Fig. 1(b)], we examined how the SL film responded to local excitations by applying voltage pulses of different amplitudes and durations. Three types of SL responses were identified by their switching threshold voltages and switching delays in time-resolved measurements. In certain SL regions, the switching behaviour supports an electric-field activated conductance change at a pulse voltage below 2.3 V, while a heat-assisted transition becomes dominant at a pulse voltage above 2.5 V and high current.

## Results

**Figure 1:**
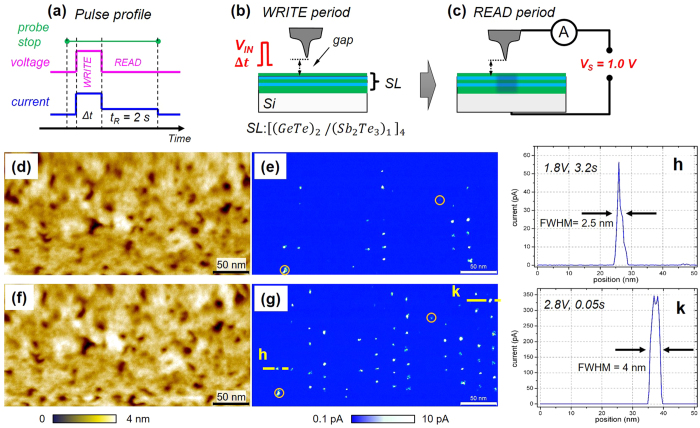
SPM topographs and conductance maps. (**a**) Profiles of a voltage pulse and an SPM current during SPL patterning. (**b**,**c**), illustration of SPM current measurements and the SL state (**b**) before and (**c**) after conductance switching in a grain beneath the SPM probe tip. (**d**,**e**), SPM topograph and the corresponding current map (set-point: Δf = 1.8 Hz, dz = 150 pm, V_S_ = +1.0 V) after voltage pulse excitations in 42 probe positions. (**f**,**g**), same as (**d**,**e**) after excitations in 84 positions. (**h**,**k**), line profiles of current maps at +1.0 V at positions indicated in (**g**), the corresponding excitation voltage and duration are indicated. Circles in (**e**,**g**) mark initial defects.

Figure 1 shows the results of two consecutive sessions of SPL patterning. We used a voltage pulse profile consisting of WRITE and READ periods as shown in Fig. 1(a). In the current map in Fig. 1(g), the pulse amplitude (V_IN_) in the WRITE period was increased from 1.8 V, the left-most column, to 2.8 V, the right-most column. Each column had a set of 7 stop points that were spatially separated by 20 nm, and the pulse duration, Δt, increased from 0.05 s, the top row, to 3.2 s, the bottom row. Six columns of current spots observed in Fig. 1(e) and 12 columns in Fig. 1(g) were created by a sequential application of the 7-stop point SPL pattern. Although changes in the film morphology in Fig. 1(d,f) were insignificant after the SPL patterning sessions, the conductance change of the SL appeared as high current spots produced by the voltage pulses with V_IN_ ≥ 1.8 V. The HCS spots created had a diameter of less than 5 nm, as shown in Fig. 1(h,k). The HCS current value of 60–320 pA was ~300 times larger than the LCS current value. However, certain areas, such as those in the middle of Fig. 1(e) and on the right-hand side of Fig. 1(g), showed no conductance switching even after excitation at V_IN_ > 2.5 V. Moreover, there was an unclear correlation among the HCS current values and V_IN_ and Δt, indicating that other factors play a role in the variations observed in the switching voltage. Recently, formations of crystal Sb_2_Ge_x_Te_3+x_ phases with x = 1, 2, 3 and 4 during the SL growth have been reported in ref. 18. Although we used different growth process^17^, a certain degree of atomic mixing and internal stress may occur in the SL, causing position-dependent variations in the switching voltage and the HCS current.

**Figure 2:**
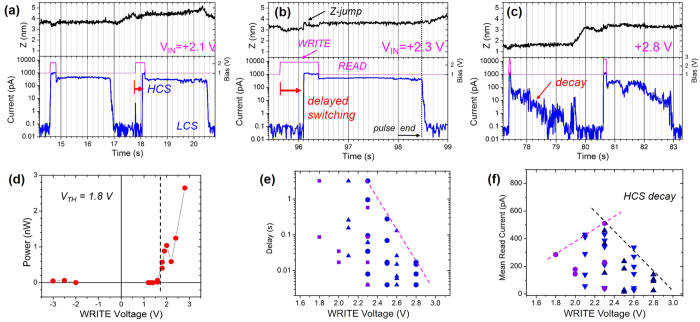
Time variation of SPM current during SPL patterning with WRITE pulses. (**a–c**) Fragments of time traces of the probe height (top panel, black), sample voltage (middle panel, pink) and SPM current (bottom panel, blue) recorded during SPL sessions with WRITE pulse voltage and duration: (**a**) 2.1 V, 200 ms and 400 ms, (**b**) 2.3 V, 800 ms, and (**c**) 2.8 V, 50 ms and 100 ms. (**d–f**), Mean switching power, switching delay, and average READ current as a function of WRITE pulse voltage. Different symbols correspond to different SPL sessions. Lines are eye guides.

A position-dependent conductance change was observed in time-dependent variations of the SPM current during the SPL patterning sessions. Fragments of recorded data during the SPL patterning sessions at *V*_*IN*_ = 2.1, 2.3 and 2.8 V, as shown in Fig. 2(a–c), illustrate characteristic features of the conductance switching behaviour. The grain-dependent features are

- Some areas showed no conductance switching, with negligible excitation current (the WRITE period) at V_IN_ < 2.3 V, as shown on the left-hand side of the current map in Fig. 1(g).
- The post-excitation current (the READ period) showed two values at 1.0 V: high (~330 pA) and low (~50 pA).
- A steep increase in the injection current accompanied an expansion of the film, which appeared as an ~0.5 nm Z-jump similar to what is observed at 96 s in Fig. 2(b). The Z-jump value was between 0.05 and 0.7 nm depending on the probe position. On average, it was 0.35 ± 0.05 nm at V_IN_ = 2.1–2.8 V, corresponding to a film expansion of ~3.4% in our ~10-nm-thick film.
- There is a time delay (*τ*) for the rise in the excitation current (the WRITE period) observed at 18 s and 96 s in Fig. 2(a,b), and the time delay is summarised in Fig. 2(e). An abrupt change in the SL current usually appeared at V_IN_ = 2.0 V and *τ*> 0.8 s, at V_IN_ = 2.1 V and *τ* > 0.2 s, and at V_IN_ = 2.8 V and *τ* ~ 0.05 s.
- The HCS current decayed upon excitation at V_IN_ ≥ 2.5 V. Small values of the mean READ current were obtained at 2.5–2.8 V because the HCS current decays in ~2 s, as shown in Fig. 2(f). By fitting the HCS current to an exponential decay function, we obtained a decay time, for an initial time of 2 s after the WRITE pulse, that is 0.1–2 s at V_IN_ = 2.8 V and is longer than 10 s at V_IN_ = 2.1 V.
- The observed HCS spots were measured at V_S_ = 1.0 V, and the measurements could be reproduced for at least 2 days.

Under pulse excitation, the SL conductance switching occurred above a threshold voltage of V_IN_ = 1.8 V. For a *steady* HCS current, the minimum electric energy required was ~90 pJ at V_IN_ = 2.1 V, ~20 pJ at V_IN_ = 2.3 V, and ~5 pJ at V_IN_ = 2.6 V, which is comparable to a reported value of 11 pJ for high-quality PCM devices based on repeated blocks of [(GeTe)_4_/(Sb_2_Te_3_)_2_] SLs in ref. 7. Fast decay of the HCS current was observed after excitation with an electric energy larger than 1 nJ at V_IN_ = 2.1 V, and 200 pJ at V_IN_ = 2.8 V, as shown in Fig. 2(c). In separate measurements with continuous (~50 s) excitation, the threshold switching voltage was +1.6 V for some areas, and it exhibited a strongly bias-polarity dependence, as shown in Fig. 2(d). A minimum injection energy of ~100 pJ was obtained for conductance switching. The values are consistent with a switching voltage of 1.4 V reported for 400-nm-PCM devices^10^ and an SET-RESET voltage of 1.2 V reported for [(GeTe)_2_/(Sb_2_Te_3_)_5_]_8_ SLs grown on a tungsten film^11^. The threshold switching voltage depends on the transition energy of Ge atoms between bonding states in the tetrahedral and octahedral sites, and the activation barrier was predicted to be 2.56 eV and 3.10 eV for two transition routes between the Ferro and the inverse Petrov structure models, corresponding to the HCS and the LCS, respectively^15^. Additionally, the threshold voltage is affected by the residual SL strain where the Ge atoms experience different displacement forces due to the mismatch of the in-plane lattice constant of GeTe and Sb_2_Te_3_ layers, different degrees of Ge/Sb mixing and changes to the layer thickness^21^.

**Figure 3:**
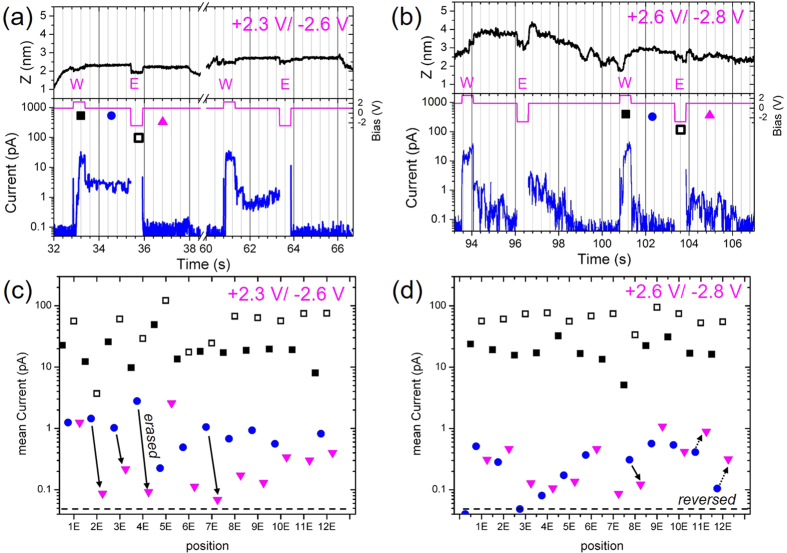
Time variation of SPM current during SPL patterning with WRITE-ERASE pulses. (**a–b**) Fragments of time traces of the probe height (top panel, black), bias voltage (middle panel, pink) and current (bottom panel, blue) recorded during SPL sessions. (**c,d**), Mean values of current for different probe positions: WRITE current (closed squares), ERASE current (open squares), READ current before (blue circles) and after (pink triangles) ERASE pulse. Mean READ current was averaged over 2 s. WRITE/ERASE pulse voltages were +2.3 V/−2.6 V in (**a**,**c**) and +2.6 V/−2.8 V in (**b,d**) and a pulse duration of 500 ms. Solid arrows marked as “erased” indicate successful HCS-to-LCS transitions.

The reversible switching was confirmed in separate measurements where an ERASE voltage pulse of negative polarity and a second READ period were added at each stop point, resulting in a WRITE-ERASE pattern. A WRITE voltage of +2.3 V and a time duration of 500 ms were selected as optimal parameters for the SL conductance switching with an injection energy of 15–100 pJ. An ERASE voltage of −2.6 V and a duration of 500 ms were used to create sufficiently strong electric field reversal. The typical behaviour of the current response is shown in Fig. 3(a), where the current changed from the LCS to HCS values and reversed backward. A steady HCS current of ~50 pA was observed at 33.5–35.5 s in Fig. 3(a) after the WRITE voltage pulse, and an LCS current of ~0.1 pA was measured again after the ERASE voltage pulse at 36 s. Such reversible switching was recorded for 5 out of 12 stop points separated by ~40 nm distance, as indicated in Fig. 3(c).

Different switching behaviour was observed at WRITE (ERASE) pulse amplitudes of +2.6 V (−2.8 V). The HCS current appeared and decayed within ~2 s after both WRITE and ERASE voltage pulses in Fig. 3(b). The mean current values were similar for the first and second READ periods shown in Fig. 3(d) even when the ERASE current was larger than the WRITE current. The dynamic behaviour of the HCS current in Fig. 3(b) resembles that in Fig. 2(c), indicating a common mechanism for the unsteady conductance state. The switching behaviour in Fig. 3 for the second sample is similar to that in Fig. 2, while smaller injection and HCS currents were measured for the second sample with a large substrate series resistance.

The fact that the HCS current appeared at both positive and negative voltage polarity indicates that two similar HCSs were created under the strong electric field from the SPM tip normal to the SL film surface.

## Discussion

The results obtained suggest that different factors contribute to the variability observed in the conductance state switching of the [(GeTe)_2_/(Sb_2_Te_3_)_1_]_4_ SLs. Geometrical reordering of atoms in the SL layers results in a change in the SL volume. The fact that the SL suddenly expands by ~3.4% upon conductance state switching, as shown in Fig. 2(b), illustrates the relationship between the atomic arrangement in the SL layers and the different SL conductance states predicted theoretically^13^,^15^. The expansion of the film in the direction normal to the film surface appeared as a sudden change in the probe Z-position, the Z-jump. As the probe-sample gap was maintained by keeping the total force acting on the probe constant, the probe approached the SL surface (Z decreased) to correct for changes in the electrostatic force upon the application of WRITE and ERASE pulses, as shown in Figs (2a,b and 3a). Such force modulation by the application of external voltage is the basic effect employed in electric potential measurements on semiconductor surfaces^22^. In addition, a sudden Z-jump, similar to what appeared during the WRITE pulse period at 96.2 s in Fig. 2(b), occurred and coincided with an abrupt current change. Usually, thermal drift causes a gradual change in the Z position, and it was negligible (~10 nm per hour), as observed in the READ periods. Therefore, a Z-jump appeared as a response to the SL expansion upon the conductance state transition. The observed expansion of ~3% is close to a reported value of ~2% for the transition of the Ge atom from 4-fold sites in the LCS to 6-fold sites in the HCS due to the difference in the Ge-Te bond length^10^. The calculated change in the lattice parameter in the direction normal to the crystal layers has been reported as being between +7.5% and −4.5% depending on the SL structure model^15^. In contrast, the volume decreases by ~6% during the phase transition from amorphous LCSs to crystalline HCSs^10^,^23^. Our results showed SL expansion during the LCS-to-HCS transition, which agrees with the solid-solid transition mechanism.

Variations in the observed responses indicate that the SL film is not uniform. From observed switching dynamics, we classified the SL responses into three groups. The main group (~52%), referred to as group C1, showed a LCS-to-HCS transition with a delay shorter than 6 ms (the time resolution in our setup). A long (0.1–3 s) delay in the conductance state transition was observed in 40% of the responses, referred to as group C2. The remaining part (~8%), referred to as group C3, showed no conductance switching below *V*_*IN*_ ~2.8 V. A large fraction of the SL responses (group C2) showed a delay in the conductance switching, a behaviour that is observed in ferroelectric materials where a persistent polarisation produced by an external electric field controls the charge transport properties. The delay time varied between ~3 s at V_IN_ = 2.1 V and 5 × 10^−3 ^s at 2.8 V. The presence of the delay in the current switching provides evidence that the WRITE current is unlikely to be the primary *trigger* for the conductance change in group C2. Alternatively, a strong electric field from the probe tip may induce electric polarisation of the SL. The electric field applied was estimated using a capacitor model including the vacuum gap and the SL film. A minimum vacuum gap of 0.44 nm was estimated from a sum of the inter-atomic distance in the top Sb_2_Te_3_ layer (0.288 nm at room temperature^24^) and the probe vibration amplitude (0.15 nm) according to ref. 25. Assuming negligible resistivity of the substrate, a gap width of 0.44 nm and a relative permittivity of 36.2 for Ge_1_Sb_2_Te_4_ films^26^, the electric field applied was 0.08–0.1 × 10^6 ^V/cm at 2.1–2.8 V, which is comparable to the coercive field of ferroelectric materials^27^,^28^. The typical polarisation time in ferroelectric films depends on the electric field strength and the temperature and is approximately 10^−3^–10^0 ^s at room temperature^28^. The observed reduction in the delay time with an increase in the excitation pulse voltage, as shown in Fig. 2(e), resembles the polarisation behaviour of ferroelectric films. Therefore, it is likely that some regions of the SL film (C2 group) possess a structure described by the ferroelectric model in ref. 10 because it is an energetically favourable geometric structure in our growth process at a temperature of 230 °C. Variations in the delay time and the HCS current value may occur because of a non-uniform mixing of Ge and Sb atoms in adjacent layers. To the best of our knowledge, the relationship between atomic mixing in the constitutive SL layers and conductance switching behaviour has not previously been reported.

The excitation voltage and the excitation current are two competing factors in conductance state switching. A *steady* HCS current after excitation at 2.1 and 2.3 V is shown in Fig. 2(a,b). In contrast, the HCS current was unsteady after excitation at 2.8 V, as shown in Fig. 2(c,b). At V_IN_ > 3.0 V, a regrowth of SL grains has been reported in ref. 29. These facts indicate that the *unsteady* HCS current was caused by excessive heating because of the hot electrons injected from the SPM tip at *V*_*IN*_ ~2.8 V and the injected electric energy above 200 pJ. Alternatively, at *V*_*IN*_ ~2.3 *V*, the *steady* HCS current in Figs (2b and 3a) indicates the balance between electronic excitation and current heating. At a pulse voltage of 2.1–2.3 V, a long delay time of ~3 s was necessary to complete the internal SL structure transformation before the conductance change. We note that the observed delay in the conductance switching in Fig. 2(e) contradicts the traditional switching model in which the Joule heat produced by electric current is the primary trigger of the conductance state switching between amorphous and crystalline states^4^,^5^. Instead, the observed response of the SL to pulse excitation supports another switching mechanism in which the crystalline phase transition is caused by a strong electric field and assisted by electric current heating.

The obtained data suggest the following mechanism for conductance switching. When the pulse voltage is above the switching threshold, high-energy electrons were injected into the SL from the SPM probe through the vacuum gap. The injected energetic electrons cause (i) excitation of atomic bonds and (ii) generation of phonons, which in turn activate atom re-bonding and displacement under the strong electric field beneath the SPM probe. An optimal crystal structure was achieved either in the short period of time (for group C1) when a strong electric field of ~0.1 MV/cm and a high electron current were applied or after the time delay required to complete the structural transition at a low electric field (for group C2). An increase in the transverse electric conduction by ~300-fold and an expansion of the SL film are the manifestations of significant structural changes during the LCS-to-HCS transition. We assume that the transition occurs on a short time scale (<1 ms) for group C1, although we are unable to resolve it with our time resolution. At a high voltage and high current injection, the phonon generation process prevails, leading to excessive heating of the SL grain and causing the unsteady HCS current shown in Fig. 3(f) and regrowth of the SL grain under V_IN_ >3.0 V in ref. 29. The observation of the reversible change of the conductance states in Fig. 3 excludes a defect-related origin of the HCS current.

In conclusion, HCS current values and a different switching dynamic were observed for individual [(GeTe)_2_/(Sb_2_Te_3_)_1_]_4_ SLs grains by employing SPM and SPL techniques. Three groups of SL responses were recognised by their behaviour under voltage pulse excitation. The HCS current delay observed in some areas of the SL film provides evidence that the strong electric field applied causes long-lasting changes in the SL structure. The data emphasise the role of the electric field in local conductance switching of the SL films.

## Methods

SL [(GeTe)_2_/(Sb_2_Te_3_)_1_]_4_ films were grown on Si(100) substrates by a two-step process that involved using an Ar-ion sputtering system with a base pressure of 1.8 × 10^−4 ^Pa. Reverse sputtering of a 3′ Si substrate was performed at a power of 100 W with an Ar flow rate of 10 sccm. The growth process included the following: (1) the deposition of a 3-nm-thick Sb_2_Te_3_ seed layer at room temperature and subsequent annealing at 230 °C, and (2) the deposition of 4 periods of GeTe(0.8 nm)/Sb_2_Te_3_(1 nm) by repeated sputtering of GeTe and Sb_2_Te_3_ alloy targets at a pressure of 0.5 Pa, with an Ar flow rate of 10 sccm, a power of 20 W and a substrate temperature of 230 °C^17^. The large (20–100 nm) grain size and uniform crystal orientation were confirmed by X-ray analysis^17^.

For SPM measurements, an Omicron SPM operating in an ultra-high vacuum chamber (~1 × 10^−7 ^Pa) at room temperature was used^22^,^25^,^29^. SPM topographs and current maps were acquired simultaneously in the constant force mode at Vs = 1.0 V and Δf = 1.8 Hz. For SPL patterning, at a specific SPM probe position, a voltage pulse was applied between a tungsten probe tip and the sample while the feedback circuit maintained the pre-defined probe-sample force. The force was specified as a shift in the resonance frequency (Δf) of a quartz length extension resonator (qLER) operating at a resonance frequency of ~1 MHz and a vibration amplitude of 150 pm^22^,^25^,^29^,^30^,^31^. Voltage pulses with an amplitude (V_IN_) of 1.0–2.9 V and a duration (Δt) of 0.05–3.2 s were used; the voltage was applied to the sample. Time-dependent changes in the SPM probe height, sample voltage and SPM current were recorded with a time interval of 6 ms.

## Additional Information

**How to cite this article**: Bolotov, L. *et al*. Morphology and Electric Conductance Change Induced by Voltage Pulse Excitation in (GeTe)_2_/Sb_2_Te_3_ Superlattices. *Sci. Rep.* **6**, 33223; doi: 10.1038/srep33223 (2016).
